# Sport specialization predicts athletic identity exclusivity in youth athletes: a three-wave RI-CLPM study of controlling coaching and non-sport peer restriction with implications for adolescent care

**DOI:** 10.3389/fpsyg.2026.1933284

**Published:** 2026-09-18

**Authors:** Xishan Liu, Hao Liu, Feng Shao, Dongdong Ma

**Affiliations:** 1 School of Physical Education, Nanyang Normal University, Nanyang, Henan, China; 2 Department of Physical Education and Arts, Xuzhou Vocational College of Industrial Technology, Xuzhou, Jiangsu, China; 3 School of Nursing, Shandong First Medical University & Shandong Academy of Medical Sciences, Tai’an, Shandong, China; 4 Department of Physical Education, Tianjin Medical University, Tianjin, China

**Keywords:** adolescent development, athletic identity exclusivity, controlling coach behaviors, non-sport peer restriction, random-intercept cross-lagged panel model, sport specialization, youth sport

## Abstract

**Background:**

Sport specialization has usually been examined through biomedical outcomes such as overuse injury, burnout, and premature dropout. Less is known about whether specialization is prospectively associated with adolescent identity development by narrowing opportunities for exploration beyond sport. Drawing on identity status theory, self-determination theory, and bioecological systems theory, this study examined whether sport specialization intensity predicts athletic identity exclusivity among youth athletes and whether longitudinal indirect pathways involve controlling coach behaviors and non-sport peer restriction.

**Methods:**

A three-wave longitudinal design was implemented across a six-month period. Participants were 542 adolescent athletes (mean age = 14.63 years, SD = 1.48; 53.5% male) recruited from youth sport academies, specialized sport schools, and elite club programs in Eastern China. Measures of sport specialization intensity, controlling coach behaviors, non-sport peer restriction, and athletic identity exclusivity were administered in September 2025 (T1), December 2025 (T2), and March 2026 (T3). Data were analyzed using a random-intercept cross-lagged panel model (RI-CLPM), which separates stable between-person differences from within-person change.

**Results:**

At the within-person level, increases in sport specialization intensity predicted subsequent increases in controlling coach behaviors, non-sport peer restriction, and athletic identity exclusivity. Controlling coach behaviors and non-sport peer restriction each predicted later identity exclusivity, and both longitudinal indirect effects were significant. Significant reciprocal paths were also found from controlling coach behaviors to subsequent specialization, suggesting a reinforcing cycle between specialized participation and controlling interpersonal climates. The RI-CLPM showed substantially better fit than the traditional cross-lagged panel model.

**Conclusion:**

Sport specialization intensity was prospectively associated with subsequent athletic identity exclusivity through longitudinal indirect pathways involving controlling coaching and non-sport peer restriction. The findings extend the literature beyond injury and burnout by identifying identity narrowing as a developmentally relevant psychosocial outcome. Youth sport programs should avoid unnecessarily intensive year-round single-sport structures, promote autonomy-supportive coaching, and preserve opportunities for non-sport peer engagement.

## 1 Introduction

Competitive youth sport has changed substantially over the past several decades. In many national sport development structures, the developmental pathway that was once characterized by diversified participation and gradual skill refinement has increasingly shifted toward intensive, year-round involvement in a single sport during childhood or early adolescence. This pattern is commonly described as early sport specialization. In this article, national sport development structures refer to the organizational arrangements through which young athletes are recruited, trained, selected, and advanced. Although definitions vary across disciplines, specialization generally involves three interrelated features: intensive training in one sport, participation in that sport for most of the year, and the exclusion or substantial reduction of alternative sport and recreational activities (Jayanthi et al., 2013; LaPrade et al., 2016). The appeal of specialization is understandable. Coaches, parents, and sport organizations often assume that early accumulation of sport-specific practice is necessary for later elite performance, an assumption frequently linked to the deliberate practice framework proposed by Ericsson et al. (1993). Yet empirical work on talent development has consistently suggested a more complicated picture. Retrospective athlete-development studies and developmental models indicate that successful athlete development often involves sampling, play, and diversification before later investment in one sport, rather than early restriction to a single pathway (Baker et al., 2003; Côté, 1999; Côté et al., 2009; Côté and Vierimaa, 2014).

The potential costs of early sport specialization have been documented most extensively in sports medicine. Consensus statements, clinical reports, and systematic scoping reviews (e.g., DiSanti and Erickson, 2019) have associated intensive single-sport training with overuse injury, overtraining, burnout, and attrition (Brenner, 2016; Feeley et al., 2016; Jayanthi et al., 2013; LaPrade et al., 2016). Psychological and youth development literature has reached similar conclusions, emphasizing that sport trajectories are embedded in family, peer, educational, and institutional contexts, rather than being merely the product of training volume (Fraser-Thomas et al., 2008; Gould et al., 1996; Stambulova et al., 2009). Nevertheless, most empirical attention has focused on physical risk, performance outcomes, or reduced intrinsic motivation, burnout, and sport dropout. Less is known about whether sport specialization shapes the broader organization of the adolescent self. This gap is important because adolescence is not only a period of athletic development; it is also a critical phase of identity exploration, social role experimentation, and future planning.

Identity status theory provides a useful lens for understanding the developmental implications of specialization. Marcia (1966, 1980) proposed that identity development involves the interplay between exploration and commitment. Healthy identity formation typically requires the opportunity to explore multiple roles, values, and future possibilities before making stable commitments. Identity foreclosure occurs when commitment is made without sufficient exploration. In sport, foreclosure can take a distinctly athletic form: the athlete becomes committed to the athletic role while alternative identities remain underdeveloped. Athletic identity is not inherently maladaptive; indeed, a strong athletic identity may support persistence, discipline, and belonging (Brewer et al., 1993). However, when athletic identity becomes exclusive, adolescents may become vulnerable to distress during injury, deselection, underperformance, or career transition (Houle et al., 2010; Lally, 2007; Murphy et al., 1996; Wylleman and Lavallee, 2004). Studies of elite youth football and former college athletes similarly show that athlete identity can become restrictive when it leaves little room for other roles and future selves (Mitchell et al., 2014; Reifsteck et al., 2016).

The present study focuses on athletic identity exclusivity. Athletic identity exclusivity is treated here as an observable indicator of identity narrowing that may be relevant to foreclosure theory, rather than as a direct assessment of Marcia’s identity foreclosure status. Specialization may promote identity exclusivity in at least two ways. First, specialization structurally limits exploration. A young athlete who trains year-round, travels frequently for competition, and spends most free time in sport settings has fewer opportunities to test alternative roles in school, community, arts, informal play, diverse non-sport peer networks, or other sports. Second, specialization may create powerful social expectations. Once an adolescent is identified as a specialized athlete, parents, coaches, peers, and institutions may reinforce the idea that continued commitment is obligatory, desirable, or even irreversible. Thus, specialization may narrow identity not only through time allocation but also through the meanings attached to athletic investment.

A further reason for focusing on identity narrowing is that youth sport participation is often justified through developmental language. Organized sport is expected to teach discipline, teamwork, perseverance, social responsibility, and psychological resilience. These benefits are most likely to occur when sport provides varied roles, supportive relationships, and opportunities for self-directed growth. By contrast, a sport pathway that is highly specialized, adult controlled, and socially restrictive may deliver performance experiences while reducing the very developmental breadth that makes youth sport valuable. From this perspective, specialization is not simply a question of whether single-sport training improves or undermines performance. It is also a question of whether the adolescent’s broader developmental ecology remains sufficiently open for exploration. This distinction aligns with developmental models that emphasize sampling, play, and progressively increasing investment rather than premature role closure (Côté et al., 2009; Côté and Vierimaa, 2014; Fraser-Thomas et al., 2008).

Bioecological systems theory emphasizes that adolescent development occurs through repeated interactions between the developing individual and the proximal contexts in which that individual participates (Bronfenbrenner, 1979, 1992). In youth sport, two proximal contexts are particularly salient: the coach-athlete relationship and the peer network. These contexts are not simply background conditions. Coaches define training climates, control selection and feedback, and often shape athletes’ sense of competence, autonomy, and future possibility. Peers provide social comparison, emotional support, role models, and opportunities for affiliation outside adult-controlled performance structures. Consequently, sport specialization may affect identity exclusivity through changes in these social environments.

The first proposed pathway involves controlling coach behaviors. Self-determination theory argues that human functioning depends on the satisfaction of the basic psychological needs for autonomy, competence, and relatedness (Deci and Ryan, 1985, 2000; Ryan and Deci, 2000). Coaching climates are central to need satisfaction in sport. Autonomy-supportive coaches provide rationale, acknowledge athletes’ perspectives, and encourage volitional engagement (Mageau and Vallerand, 2003). In contrast, controlling coaches rely on intimidation, negative conditional regard, coercive rewards, excessive personal control, and pressure to comply (Bartholomew et al., 2010, 2011a). Such behaviors have been linked to need thwarting and diminished well-being in sport (Bartholomew et al., 2011b; Blanchard et al., 2009). Specialization may increase exposure to controlling coaching because specialized programs are often performance oriented, hierarchical, and outcome driven. The specialized athlete may be treated as an investment whose value must be protected through compliance, discipline, and constant performance. Under these conditions, the athlete may internalize a narrowed identity: being an athlete becomes not one domain of life but the condition for approval, belonging, and perceived worth.

The second proposed pathway involves non-sport peer restriction. Developmental sport research has long recognized that peers contribute to motivation, enjoyment, competence beliefs, and psychosocial adjustment (Holt et al., 2008; Smith, 1999; Ullrich-French and Smith, 2006; Weiss and Stuntz, 2004). Peer relationships are especially important during adolescence because they provide varied feedback and allow young people to try multiple identities in different social settings. Intensive specialization can reduce these opportunities. Athletes may miss school activities, informal gatherings, community events, and diversified peer groups because of training, travel, and recovery demands. The social world of the athlete may become increasingly restricted to teammates, coaches, and sport-centered peers. Although sport peers can provide valuable support, a peer network confined largely to sport may also reinforce identity narrowing by making the athletic role the central basis of social belonging.

Empirically, most research on specialization and psychosocial outcomes has relied on cross-sectional designs. Cross-sectional evidence is valuable for mapping associations, but it cannot clarify temporal ordering. It remains unclear whether specialization predicts later identity exclusivity, whether exclusive athletic identity leads adolescents to specialize more intensely, or whether stable third variables such as achievement orientation, parental expectations, or sport culture explain both. Longitudinal designs are therefore necessary. However, traditional cross-lagged panel models (CLPMs) also have limitations because they conflate stable between-person differences with within-person change. Hamaker et al. (2015) showed that cross-lagged effects from a conventional CLPM may not represent intra-individual processes when stable between-person heterogeneity is present. The random intercept cross-lagged panel model (RI-CLPM) addresses this problem by separating time-invariant between-person differences from dynamic within-person deviations. The approach has become increasingly important in developmental and psychological research, though it still requires careful interpretation and transparent reporting (Hamaker et al., 2015; Mulder and Hamaker, 2021).

The present study uses a three-wave longitudinal design and an RI-CLPM to examine the within-person association between sport specialization intensity and athletic identity exclusivity across a competitive season. We advance four hypotheses. First, within-person increases in sport specialization will predict subsequent increases in athletic identity exclusivity. Second, controlling coach behaviors will mediate this longitudinal association. Third, non-sport peer restriction will also mediate this longitudinal association. Fourth, controlling coach behaviors and sport specialization will show reciprocal within-person effects, such that more controlling coaching predicts later increases in specialization and specialization predicts later increases in controlling coaching. Figure 1 presents the hypothesized model.

**Figure 1:**
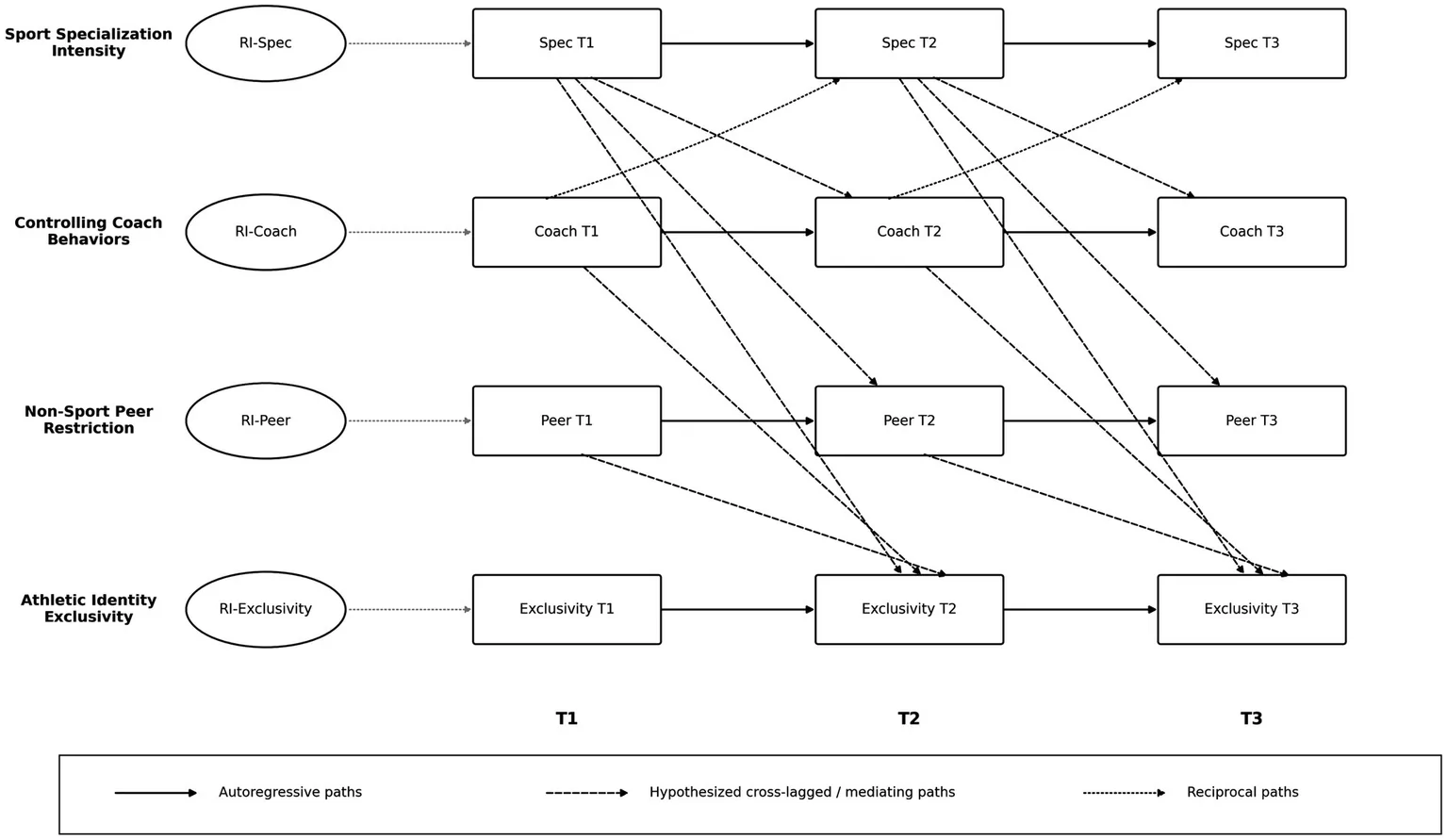
Hypothesized random-intercept cross-lagged panel model. RI = random intercept. Within-wave covariances were estimated but are omitted for clarity.

## 2 Method

### 2.1 Participants and procedure

Participants were recruited from regional youth sport academies, specialized sport schools, and elite club programs in a large metropolitan area in Eastern China. Programs were eligible if they provided organized competitive training for adolescents and required at least three structured training sessions per week. Athletes were eligible if they were 12 to 17 years old, currently enrolled in one of the participating programs, and actively involved in organized competition. The final sample represented five sport categories: gymnastics (*n* = 112), swimming (*n* = 105), soccer (*n* = 120), basketball (*n* = 118), and tennis (*n* = 87). The five sport categories reflected the regional programs available through convenience sampling; together, they included individual and team sports with different typical specialization timelines and peer structures.

A total of 750 athletes were initially invited to participate. Written informed consent was obtained from legal guardians, and assent was obtained from athletes before data collection. To protect anonymity while enabling longitudinal matching, participants generated a self-identifying alphanumeric code based on stable personal prompts that did not directly reveal their identity. Data were collected in September 2025 (T1), December 2025 (T2), and March 2026 (T3). Because competition calendars differed across the five sports, the waves are described according to elapsed time rather than as uniform competitive phases.

Valid baseline questionnaires were returned by 680 athletes recruited across 14 regional sport programs. Attrition over the study period was attributed primarily to injury, program transfer, and voluntary withdrawal. Specifically, 605 athletes completed the T2 assessment, and the final analytic sample consisted of 542 athletes who provided complete matched data across all three waves. The sample included 290 males (53.5%) and 252 females (46.5%). At T1, the mean age was 14.63 years (SD = 1.48), mean sport tenure was 6.24 years (SD = 2.15), and mean weekly training volume was 14.5 h (SD = 4.2). Table 1 presents sample characteristics and descriptive statistics for the primary constructs.

**Table 1:** Sample characteristics and descriptive statistics for primary study variables.

Variable	T1 M (SD)	T2 M (SD)	T3 M (SD)	Reliability range (α)
Age (years)	14.63 (1.48)	—	—	—
Sport tenure (years)	6.24 (2.15)	—	—	—
Weekly training volume (hours)	14.5 (4.2)	—	—	—
Sport specialization intensity	3.42 (0.88)	3.51 (0.85)	3.55 (0.87)	0.86–0.88
Controlling coach behaviors	3.75 (1.12)	3.88 (1.15)	4.02 (1.18)	0.90–0.93
Non-sport peer restriction	2.95 (0.94)	3.12 (0.96)	3.25 (1.02)	0.82–0.85
Athletic identity exclusivity	4.10 (1.25)	4.35 (1.30)	4.52 (1.35)	0.88–0.90

### 2.2 Measures

All measures were administered in Simplified Chinese. Instruments not previously available in that language were translated using a standard forward-translation and back-translation procedure. Two independent bilingual psychology researchers performed the initial forward translation, while two other bilingual researchers, who were blind to the original English versions, conducted the back-translation. Differences were resolved through consensus by a third bilingual researcher. A pilot administration with 20 adolescent athletes resulted in minor wording adjustments for clarity. Unless otherwise indicated, scale scores were computed as item means, with higher scores indicating higher levels of the construct.

Sport specialization intensity. Sport specialization intensity was assessed using a researcher-developed, five-item continuous measure based on the clinical criteria and conceptual definitions of specialization (Brenner, 2016; Jayanthi et al., 2013; LaPrade et al., 2016). Rather than using the traditional binary (yes/no) categorization, this continuous scale was designed to capture the current intensity of single-sport investment among adolescents. Items assessed intensive single-sport involvement, year-round commitment, and reduced participation in other sport or leisure activities. Example items were: “I spend almost all of my free time training for this specific sport” and “I have given up participating in other sports to focus only on this one.” Responses were made on a 5-point scale ranging from 1 (strongly disagree) to 5 (strongly agree). Internal consistency was good across waves.

Controlling coach behaviors. Athletes’ perceptions of controlling coaching were assessed using the Controlling Coach Behaviors Scale (CCBS; Bartholomew et al., 2010). We translated and administered the original 15-item, four-dimension version using a 7-point scale (1 = strongly disagree, 7 = strongly agree). Although Zhao and Zhou (2022) validated a modified Chinese short form comprising nine items, three dimensions, and a 5-point response scale, the full original version was used to retain coverage of all four theoretical facets, including intimidation. A composite score was calculated because our hypotheses focused on the overarching controlling interpersonal climate rather than specific subdimensions. Internal consistency of the composite was high across waves (α = 0.90–0.93); however, high internal consistency does not by itself establish strict unidimensionality, and the composite-score approach is acknowledged as a measurement limitation.

Non-sport peer restriction. A researcher-developed six-item scale assessed perceived sport-related restrictions in access to non-sport peers and age-typical social activities; it was not intended as a general measure of emotional loneliness. The construct was informed by youth sport peer research emphasizing the developmental value of diverse peer experiences (Holt et al., 2008; Smith, 1999). Example items were: “Because of my training schedule, I feel disconnected from friends outside my sport,” “I miss out on normal social activities that other young people my age get to do,” and “My sport commitments leave me with almost no time to participate in school clubs or non-sport events.” Responses were made on a 5-point scale from 1 (almost never) to 5 (almost always).

Athletic identity exclusivity. Athletic identity exclusivity was assessed using the exclusivity dimension of the original 10-item Athletic Identity Measurement Scale (AIMS; Brewer et al., 1993). Specifically, we utilized the three items (Items 4, 5, and 9) that capture the extent to which sport occupies a dominant or exclusive place in the athlete’s self-concept. An example item is: “Sport is the only important thing in my life.” Although some later 7-item short-forms of the AIMS omit Item 9, it was retained here for its conceptual relevance to identity narrowing. We interpret this construct as a theoretically relevant indicator of identity exclusivity, rather than a direct clinical measure of identity foreclosure (Marcia, 1966). Responses were made on a 7-point scale ranging from 1 (strongly disagree) to 7 (strongly agree). Higher scores indicated stronger athletic identity exclusivity.

### 2.3 Data analysis

Analyses proceeded in four steps. First, data were screened for out-of-range values, distributional irregularities, and missing-data patterns. Internal consistency coefficients, descriptive statistics, and bivariate correlations were computed for all study variables. Little’s (1988) test was used as a preliminary assessment of whether missingness was completely at random. Attrition checks compared athletes retained across all three waves with those who discontinued after T1. Independent-samples t tests were used for continuous baseline variables, and a chi-square test was used for gender distribution. Primary structural models were estimated using the matched complete sample (*N* = 542). Because complete-case analysis may be biased when missingness depends on unobserved variables, this issue was considered when interpreting the findings (Enders, 2010).

Second, longitudinal measurement invariance was tested before interpreting structural paths. Following recommendations from Vandenberg and Lance (2000), configural, metric, and scalar invariance models were compared. The four constructs were evaluated jointly in a single comprehensive longitudinal measurement model. To maintain an appropriate indicator-to-sample-size ratio, item parcels or dimension scores were used as indicators for the lengthier measures, including the CCBS, consistent with the model specification implemented in Mplus. Residuals of corresponding indicators were allowed to correlate across waves to account for indicator-specific stability over time. Model fit was evaluated with the comparative fit index (CFI), Tucker–Lewis index (TLI), root mean square error of approximation (RMSEA), and standardized root mean square residual (SRMR). The interpretive criteria followed Hu and Bentler (1999), while recognizing that fit indices should be interpreted jointly rather than as rigid cutoffs. Kline’s (2016) recommendations were used to guide reporting of model fit and standardized estimates.

Third, a traditional cross-lagged panel model was compared with an RI-CLPM. The traditional CLPM estimates autoregressive and cross-lagged relations among observed variables but does not separate stable between-person differences from within-person change. The RI-CLPM adds latent random intercepts for each repeated construct, with loadings fixed to unity, and estimates autoregressive and cross-lagged paths among within-person residual components (Hamaker et al., 2015; Mulder and Hamaker, 2021). This decomposition allows the cross-lagged paths to be interpreted as associations between deviations from an individual’s expected level and later deviations in another construct.

Fourth, longitudinal indirect effects were evaluated using bias-corrected bootstrapping with 5,000 resamples (Preacher and Hayes, 2008). The primary RI-CLPM was estimated using robust maximum likelihood (MLR). To obtain bias-corrected bootstrap confidence intervals for indirect effects, the same model was re-estimated using maximum likelihood (ML) with 5,000 bootstrap resamples. Because all constructs were measured via self-report, common method bias was considered in the interpretation (Podsakoff et al., 2003). Models were estimated in Mplus 8.4 (Muthén and Muthén, 1998–2017).

## 3 Results

### 3.1 Preliminary analyses and attrition check

Little’s MCAR test indicated that the missing-data pattern did not depart significantly from complete randomness, χ^2^(142) = 156.34, *p* = 0.197. Independent-samples t tests were used to compare continuous baseline variables between athletes who completed all three waves and those who discontinued after T1, while a chi-square test was used to compare gender distributions. No significant baseline differences were found in age, weekly training volume, sport tenure, sport specialization intensity, controlling coach behaviors, non-sport peer restriction, athletic identity exclusivity, or gender distribution (all ps > 0.05). Thus, we found no evidence of systematic attrition with respect to the observed baseline variables examined.

A confirmatory factor analysis (CFA) of the researcher-developed sport specialization intensity scale at T1 provided preliminary support for a one-factor structure in the present sample, χ^2^(5) = 14.25, *p* = 0.014, CFI = 0.965, TLI = 0.942, RMSEA = 0.058, 90% CI [0.027, 0.093], and SRMR = 0.041. Internal consistency was acceptable to excellent across all variables and waves. Correlations among constructs were positive and in the expected direction. Sport specialization intensity was positively associated with controlling coach behaviors, non-sport peer restriction, and athletic identity exclusivity at concurrent and subsequent waves. Controlling coach behaviors and non-sport peer restriction were also positively associated with exclusivity. These preliminary results supported the plausibility of the proposed longitudinal model, but they could not distinguish within-person change from stable between-person differences.

### 3.2 Measurement invariance and model comparison

The longitudinal CFA supported measurement invariance across the three waves. The configural model demonstrated good fit. Imposing equality constraints on factor loadings produced only a negligible decline in fit, supporting metric invariance. Further constraining item intercepts also resulted in minimal fit decrement, supporting scalar invariance. These results indicated that the constructs were measured comparably across time, allowing longitudinal change and cross-lagged relations to be interpreted meaningfully. Table 2 summarizes the measurement invariance tests.

**Table 2:** Longitudinal measurement invariance and structural model comparison.

Model	χ^2^	df	CFI	TLI	RMSEA	SRMR	ΔCFI	ΔRMSEA
Configural CFA	642.15	365	0.962	0.955	0.037	0.041	—	—
Metric CFA	668.42	389	0.959	0.954	0.036	0.044	−0.003	−0.001
Scalar CFA	694.70	413	0.957	0.953	0.036	0.046	−0.002	0.000
Traditional CLPM	285.64	44	0.912	0.885	0.098	0.075	—	—
RI-CLPM	45.32	28	0.988	0.975	0.034	0.025	—	—

The RI-CLPM was then compared with a traditional CLPM. The traditional CLPM showed weaker descriptive fit, whereas the RI-CLPM showed excellent fit. The markedly improved CFI, TLI, RMSEA, and SRMR supported using the RI-CLPM for hypothesis testing. This comparison is substantively important because stable between-person differences in specialization, coaching climate, peer restriction, and identity exclusivity should not be conflated with within-person developmental dynamics.

### 3.3 Within-person cross-lagged effects

The RI-CLPM results supported the hypothesized within-person processes. At the between-person level, random intercepts for the four constructs were positively correlated, indicating that athletes who were generally more specialized across the season also tended to report generally higher controlling coaching, peer restriction, and exclusivity. These stable associations are theoretically meaningful, but the primary hypotheses concerned within-person fluctuations around these stable tendencies.

Within-person autoregressive paths were significant for all constructs across both intervals, indicating moderate intra-individual continuity. Sport specialization intensity showed autoregressive effects from T1 to T2 and from T2 to T3. Controlling coach behaviors, non-sport peer restriction, and athletic identity exclusivity also showed significant autoregressive stability. These coefficients suggest that while the constructs were stable, there remained sufficient within-person variability to test cross-lagged effects.

Consistent with Hypothesis 1, within-person increases in sport specialization intensity predicted later increases in athletic identity exclusivity. Athletes who reported higher specialization than their own expected level at T1 subsequently reported higher exclusivity at T2, and the same pattern was observed from T2 to T3. This finding indicates that specialization is not merely correlated with identity exclusivity because some athletes are chronically high on both constructs; rather, increases in specialization over time were associated with subsequent identity narrowing.

Consistent with Hypotheses 2 and 3, sport specialization predicted increases in both proposed mediators. T1 specialization predicted T2 controlling coach behaviors and T2 non-sport peer restriction. T2 specialization similarly predicted T3 controlling coach behaviors and T3 non-sport peer restriction. Both mediators then predicted later exclusivity. Specifically, controlling coach behaviors predicted subsequent athletic identity exclusivity, and non-sport peer restriction also predicted subsequent exclusivity. These effects are consistent with an interpretation in which specialization is prospectively associated with identity development through changes in the interpersonal climate of sport and restrictions in peer life outside sport.

Hypothesis 4 was also supported. Controlling coach behaviors predicted subsequent sport specialization intensity across both intervals, indicating a reciprocal cycle. When athletes perceived their coaches as more controlling than usual, they subsequently reported higher specialization relative to their own expected level. In contrast, non-sport peer restriction did not significantly predict later specialization in the original model estimates. This suggests that the reciprocal reinforcement process may be specific to the coach-athlete authority relationship rather than to all social restrictions associated with specialization. Table 3 reports the standardized within-person path coefficients. Figure 2 presents the significant within-person paths.

**Table 3:** Standardized within-person autoregressive and cross-lagged paths from the RI-CLPM.

Path	T1 → T2 β	SE	p	T2 → T3 β	SE	*p*
Spec → Spec	0.35	0.04	< 0.001	0.32	0.04	<0.001
Coach → Coach	0.38	0.04	< 0.001	0.40	0.05	<0.001
Peer → Peer	0.31	0.05	< 0.001	0.28	0.04	<0.001
Exclusivity → Exclusivity	0.42	0.04	< 0.001	0.39	0.04	<0.001
Spec → Exclusivity	0.18	0.06	0.003	0.21	0.05	<0.001
Spec → Coach	0.24	0.05	< 0.001	0.22	0.06	<0.001
Coach → Exclusivity	0.17	0.05	0.001	0.22	0.05	<0.001
Spec → Peer	0.27	0.06	< 0.001	0.26	0.05	<0.001
Peer → Exclusivity	0.19	0.06	0.002	0.25	0.06	<0.001
Coach → Spec	0.16	0.06	0.012	0.19	0.06	0.004
Peer → Spec	0.08	0.05	0.110	0.10	0.06	0.095

**Figure 2:**
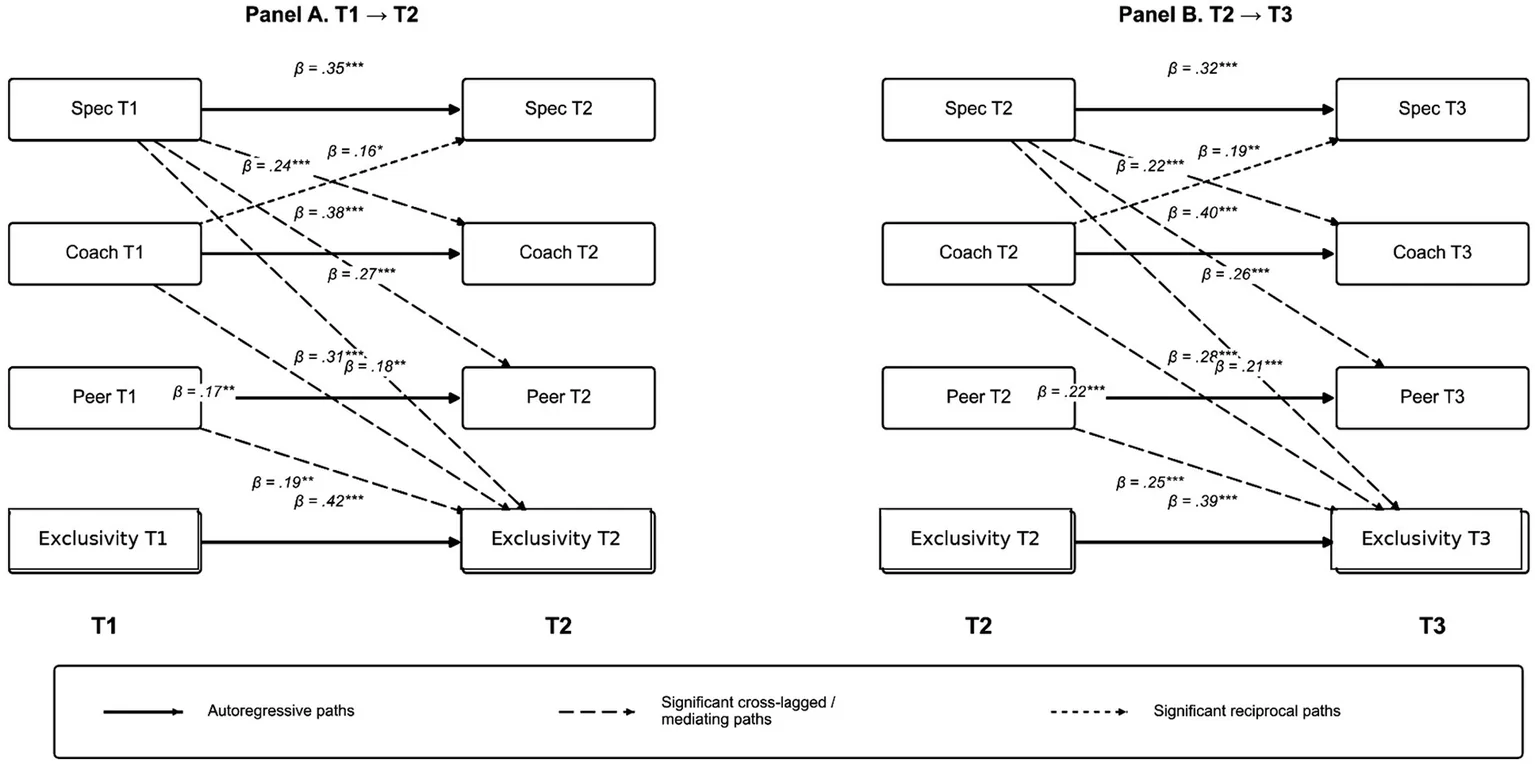
Significant within-person paths from the RI-CLPM. Only significant within-person paths are shown. Random intercepts, within-wave covariances, and nonsignificant paths are omitted for clarity. *p < 0.05, **p < 0.01, ***p < 0.001.

### 3.4 Longitudinal indirect effects

Bootstrapped indirect effects supported the proposed mediation pathways. The indirect effect from T1 sport specialization intensity to T3 athletic identity exclusivity through T2 controlling coach behaviors was significant. The indirect effect through T2 non-sport peer restriction was also significant. The total indirect effect, combining the two mediators, was significant as well. These results indicate that the longitudinal association between specialization and identity narrowing was partly explained by both social-environmental pathways.

The peer pathway had a slightly larger point estimate than the controlling-coach pathway, but the confidence intervals overlapped. Therefore, the present study does not claim that one mediator was statistically stronger than the other. A formal contrast of indirect effects would be required to make such a claim. The most defensible interpretation is that both mechanisms were meaningful and functionally distinct: one reflects the authority structure of specialized sport, and the other reflects restricted access to broader adolescent peer life (see Table 4).

**Table 4:** STDYX-standardized bootstrap estimates of longitudinal within-person indirect effects.

Indirect pathway	Estimate	SE	95% BC CI lower	95% BC CI upper
Spec T1 → Coach T2 → Exclusivity T3	0.053	0.020	0.021	0.098
Spec T1 → Peer T2 → Exclusivity T3	0.068	0.021	0.033	0.114
Total indirect effect	0.121	0.032	0.066	0.192

## 4 Discussion

### 4.1 Overview of principal findings

This study examined whether sport specialization intensity predicts athletic identity exclusivity in youth athletes and whether this association operates through controlling coach behaviors and non-sport peer restriction. Using a three-wave longitudinal design and an RI-CLPM, the study provides within-person evidence for a developmental process that has been theoretically plausible but empirically underexamined. When athletes became more specialized relative to their own expected level, they subsequently reported greater identity exclusivity. Significant longitudinal indirect associations were observed through increases in controlling coach behaviors and non-sport peer restriction. In addition, controlling coaching predicted later specialization, suggesting that specialized sport participation and controlling interpersonal climates may reinforce each other across a competitive season.

A central contribution of the study is therefore not merely that specialization predicted identity narrowing, but that the association was located within the everyday relational ecology of specialized sport. The findings suggest that identity narrowing is not best understood as a simple consequence of training hours alone. Rather, specialization appears to reorganize the developmental setting in which adolescents interpret sport, authority, friendship, and future possibility. When the sport context becomes more exclusive, the athlete may receive more messages that performance is the primary basis for recognition, while simultaneously having fewer opportunities to experience competence and belonging in non-sport domains. This interpretation is consistent with ecological and motivational perspectives because it treats identity development as a situated process rather than as an isolated individual trait (Bronfenbrenner, 1979, 1992; Deci and Ryan, 2000; Ryan and Deci, 2000). It also helps explain why the two mediators were both significant: coaches and peers represent different but complementary parts of the adolescent sport ecology.

The findings extend the specialization literature in several ways. Prior research and consensus statements have emphasized that early specialization is associated with physical injury, burnout, and dropout risk (Brenner, 2016; DiSanti and Erickson, 2019; Feeley et al., 2016; Jayanthi et al., 2013; LaPrade et al., 2016). The present study indicates that specialization may also matter for identity development. This does not mean that specialization is uniformly harmful or that strong athletic commitment is inherently problematic. Athletic identity can motivate training, promote belonging, and help adolescents organize goals. The concern is identity exclusivity: the dominance of the athletic role in self-definition. Such exclusivity may coincide with reduced opportunities to explore alternative domains, although exploration was not directly measured in the present study. This distinction is important because the same behaviors that appear adaptive in the short term, such as intensive practice and strong athletic dedication, may become developmentally constraining when they are embedded in a narrow and externally controlled life structure.

### 4.2 Sport specialization and athletic identity exclusivity

The direct within-person effect of specialization on identity exclusivity is consistent with propositions derived from identity status theory. Marcia (1966, 1980) emphasized that foreclosure occurs when commitment precedes adequate exploration. Specialization can be understood as a social and temporal condition that reduces exploration. The athlete may not merely choose sport as one meaningful domain among many; instead, sport may become the default and increasingly exclusive basis for self-definition.

Notably, specialization retained significant adjacent-wave prospective associations with later athletic identity exclusivity (*β* = 0.18 and 0.21). These remaining associations may reflect structural constraints not fully represented by the two proposed social-environmental pathways, including the sheer time devoted to training, travel, and recovery, as well as institutional expectations surrounding continued athletic investment. Because the adjacent-wave standardized paths and the two-step indirect effects do not constitute a directly comparable total-effect decomposition, their magnitudes are not compared statistically.

The RI-CLPM strengthens this interpretation by showing that increases in specialization predicted subsequent increases in exclusivity after stable between-person differences were separated from within-person dynamics. This is particularly important because adolescents who are generally more achievement-oriented or who have families with stronger sport investment may be higher on both specialization and exclusivity at all waves. The RI-CLPM does not eliminate all alternative explanations, but it provides stronger evidence for temporal within-person processes than a traditional CLPM or cross-sectional model.

### 4.3 Controlling coaching as a social-environmental pathway

The coaching pathway identifies one potential pathway through which specialization may narrow identity. Specialized athletes often train in settings where performance evaluation is frequent and selection pressure is high. In such settings, coaches may use controlling strategies to regulate effort, discipline, and compliance. Self-determination theory suggests that controlling interpersonal behaviors undermine autonomy and need satisfaction (Deci and Ryan, 1985, 2000; Ryan and Deci, 2000). In sport, the CCBS literature shows that controlling coaching includes negative conditional regard, intimidation, and excessive personal control (Bartholomew et al., 2010, 2011a). The present findings suggest that when athletes become more specialized, they may also become more exposed to or more sensitive to controlling coaching. This exposure, in turn, predicts identity exclusivity. A plausible developmental interpretation is that controlling coaching makes the athletic role feel compulsory rather than chosen. Athletes may then preserve approval and belonging by investing more exclusively in sport, thereby narrowing the self.

The reciprocal path from controlling coach behaviors to later specialization is also notable. It suggests that specialized participation and controlling coaching may form a feedback loop. A coach who uses pressure and conditional approval may intensify an athlete’s sense that sport must remain central and that alternatives are distractions or failures. In turn, greater specialization may increase a coach’s perception that the athlete is committed to high-performance goals, encouraging further control. From a developmental systems perspective, this reciprocal cycle can be understood through both socialization and selection effects. Controlling coaches may socialize athletes into highly specialized routines, but highly specialized and high-stakes sport environments may also select for or incentivize controlling coaching strategies. Therefore, this feedback loop highlights an ecosystem issue rather than merely an individual coaching style problem. Breaking this cycle may require coach education that goes beyond generic encouragement and addresses the subtle ways in which performance pressure, conditional regard, and role narrowing operate in specialized environments.

### 4.4 Non-sport peer restriction as a developmental pathway

The peer restriction pathway provides a second, distinct longitudinal pathway. Adolescents require diverse social contexts in order to test possible selves, experience different forms of recognition, and develop non-athletic competencies. Peer relationships in sport can be positive, but they are not interchangeable with non-sport friendships, school-based peer roles, and broader community participation (Holt et al., 2008; Smith, 1999; Ullrich-French and Smith, 2006; Weiss and Stuntz, 2004). When specialization restricts peer diversity, the athlete’s social feedback becomes increasingly sport centered. Teammates, coaches, and sport peers may all reinforce the same role expectations. Under these conditions, the adolescent may come to experience the athletic identity as the only socially validated identity. The significant indirect effect through non-sport peer restriction indicates that identity narrowing is not only a coaching problem; it is also a social ecology problem.

### 4.5 Reciprocal dynamics and pathway specificity

The absence of significant reciprocal effects from peer restriction to later specialization deserves attention. Non-sport peer restriction predicted later exclusivity but did not significantly predict later specialization. This pattern suggests that restricted peer life may narrow identity without necessarily increasing training commitment or single-sport investment. By contrast, coaches have direct authority over training demands, selection, and performance expectations, which may explain why controlling coaching showed reciprocal links with specialization. Bioecological systems theory helps interpret this asymmetry. Peers are developmentally powerful, but coaches in specialized sport often occupy a formal authority position that gives them direct influence over time use, performance evaluation, and opportunity structure (Bronfenbrenner, 1979, 1992). Thus, peer restriction and controlling coaching may both contribute to exclusivity but through different forms of social power.

### 4.6 Practical implications for youth sport

The findings have practical implications for youth sport policy. Recommendations to delay specialization and encourage diversified participation have often been justified on physical and performance grounds (Côté et al., 2009; Côté and Vierimaa, 2014; LaPrade et al., 2016). The present results suggest that these recommendations are also relevant to identity development. Because greater specialization intensity prospectively predicted identity exclusivity, sport organizations should consider monitoring training loads, safeguarding off-season periods, and ensuring athletes have structural opportunities for non-sport exploration. However, policy alone is unlikely to be sufficient. Some athletes may specialize later but still experience controlling coaching and peer restriction. Therefore, sport systems should address both the structure of participation and the quality of social environments. Because the study assessed current specialization intensity rather than age at specialization, the findings most directly support reducing unnecessarily intensive year-round structures rather than establishing a specific age threshold.

Coach education is a particularly important intervention target. Programs should teach coaches to distinguish high standards from controlling behavior. Autonomy-supportive coaching does not mean low expectations or lack of discipline. It means providing rationale, acknowledging athletes’ perspectives, offering meaningful choice when possible, avoiding conditional approval, and recognizing athletes as whole persons rather than only as performers (Mageau and Vallerand, 2003). In specialized contexts, coaches should actively communicate that sport excellence is compatible with education, friendships, rest, and non-sport identity exploration. This message is especially important for adolescents who may interpret coach pressure as evidence that they have no legitimate identity outside sport.

Programs should also protect peer diversity. This may involve scheduling practices so that athletes can participate in school events, discouraging unnecessary year-round commitments, facilitating non-sport group activities, and educating parents about the developmental value of ordinary peer experiences. Youth sport organizations often treat social time outside sport as a distraction from performance. The present findings suggest that such social time may instead be a protective factor against identity narrowing. Maintaining friendships outside sport can provide emotional support during injury, performance slumps, and transitions out of competitive participation.

The results also invite caution in how practitioners interpret athlete commitment. High commitment is often treated as a sign of readiness for intensified specialization. However, commitment can be qualitatively different depending on whether it is autonomous or controlled. A young athlete may train intensively because sport is meaningful, enjoyable, and personally chosen; another may train intensively because approval, selection, or belonging feels conditional on doing so. These two athletes may look similar in terms of training volume but differ sharply in identity risk. Coaches and parents should therefore ask not only how much an athlete is training, but also whether the athlete retains a sense of choice, whether non-sport identities remain available, and whether relationships outside sport are being preserved. This distinction is consistent with self-determination theory and may help reconcile why some specialized athletes thrive while others become vulnerable to burnout, distress, or identity narrowing (Deci and Ryan, 2000).

At the organizational level, the findings imply that safeguarding adolescent identity development should be treated as a quality indicator in youth sport programs. Clubs and academies commonly monitor attendance, training volume, competition results, injury status, and selection outcomes. Far fewer systems monitor whether athletes maintain friendships outside sport, whether coaches use autonomy-supportive communication, or whether athletes perceive that they have meaningful futures beyond athletic performance. These indicators are not peripheral. They are central to whether a sport environment supports development rather than only short-term performance. Programs could therefore include periodic psychosocial screening, anonymous athlete feedback about coaching climate, parent education about diversification, and scheduling policies that protect school and peer engagement. Such practices would not require lowering performance standards; rather, they would clarify that performance development and whole-person development should be pursued together.

### 4.7 Limitations and future directions

Several limitations should be acknowledged. First, all constructs were measured using self-report. Self-report is appropriate for perceptions of coaching, peer restriction, and identity, but it increases the possibility of shared method variance (Podsakoff et al., 2003). Future studies should incorporate coach reports, parent reports, observational coding of coaching behavior, and sociometric measures of peer network diversity. Second, although the design was longitudinal, the study covered one competitive season. Longer multi-year designs are needed to examine whether seasonal increases in exclusivity accumulate into more stable developmental outcomes, including distress during injury, deselection, or retirement (Lally, 2007; Stambulova et al., 2009; Wylleman and Lavallee, 2004).

Third, the sample was drawn from specialized youth sport contexts in Eastern China. The findings are most directly applicable to adolescents aged 12–17 years enrolled in competitive youth academies, specialized sport schools, and elite club programs in this metropolitan region. Unmeasured cultural factors, such as strong authority orientation, intense academic pressure, and specific elite sport structures, may influence the observed pathways. Cross-cultural replication in different sport systems is needed before generalizing the effect sizes.

Fourth, the psychometric properties of the measures present notable limitations. Both the sport specialization intensity scale and the non-sport peer restriction scale were researcher-developed measures tailored for this study. While both demonstrated good internal consistency, and the specialization scale additionally showed preliminary support for a one-factor structure at T1, neither measure has undergone the extensive cross-validation available for established instruments. The structural, convergent, and discriminant validity of the peer-restriction measure, in particular, requires further independent examination. Furthermore, regarding the CCBS, we utilized a composite score of the original 15-item version to represent the general controlling climate; this scoring choice and its underlying dimensionality warrant further structural validation in Chinese youth samples. Finally, while the three-item exclusivity subscale of the original AIMS provides a conceptually stable measure suited for this longitudinal design, it primarily assesses the dominance of the athletic role. Future studies should combine contemporary athletic identity assessments (e.g., Brewer et al., 2022) with direct multidimensional measures of identity exploration and commitment.

Fifth, the primary structural analyses used complete cases. Although the observed baseline attrition checks did not identify significant differences, estimates may still be biased if missingness was related to unmeasured variables. Future studies should use full-information maximum likelihood and report sensitivity analyses based on all available observations.

Sixth, athletes were nested within sport programs and potentially within specific coaching groups. Because complete and consistent clustering identifiers were unavailable across all participants, residual dependence at the program or coach level could not be explicitly modeled. Future multilevel studies should examine whether specialization dynamics vary significantly across different training environments.

Finally, the RI-CLPM improves on the traditional CLPM by separating stable between-person differences from within-person dynamics, but it is not equivalent to experimental causal inference. Unmeasured time-varying confounders may still influence the estimated paths.

Future research should examine moderators. The effects of specialization may vary by sport type, gender, age, socioeconomic resources, parental expectations, and competitive level. Some sports have earlier normative specialization timelines, whereas others allow later sampling. The identity consequences of specialization may also depend on whether athletes perceive specialization as self-chosen or externally imposed. In addition, future studies should investigate protective factors, such as autonomy-supportive coaching, parental emotional support, school belonging, and participation in non-sport activities. Finally, qualitative research could illuminate how athletes narrate the relationship between sport commitment and personal identity, especially during key transitions such as injury, deselection, school graduation, and movement between competitive levels.

## 5 Conclusion

This study provides longitudinal within-person evidence that increases in sport specialization intensity were prospectively associated with later increases in athletic identity exclusivity among youth athletes. Significant longitudinal indirect pathways involved controlling coach behaviors and non-sport peer restriction. A reciprocal pathway from controlling coaching to later specialization further suggests that specialized sport participation and controlling interpersonal climates may become mutually reinforcing. These findings broaden the conversation about specialization beyond injury, burnout, and dropout. For adolescents, the issue is not only how much they train, but also how specialized training is associated with autonomy, relationships, and opportunities for identity exploration. Youth sport environments that support excellence while preserving autonomy, peer diversity, and multidimensional identity development may be more developmentally sustainable.

## Data Availability

The raw data supporting the conclusions of this article will be made available by the authors, without undue reservation.
